# Behavioral and psychosocial predictors of e-cigarette use in rural Thailand: A cross-sectional propensity score-matched study

**DOI:** 10.18332/tid/218287

**Published:** 2026-05-08

**Authors:** Jatuporn Sorasit, Wuttiphong Phakdeekul, Warinmad Kedthongma

**Affiliations:** 1 Faculty of Public Health, Kasetsart University, Sakon Nakhon, Thailand

**Keywords:** e-cigarette determinants, propensity score methods, rural Thailand, alcohol consumption, regulatory compliance

## Abstract

**INTRODUCTION:**

E-cigarette use in rural Thailand persists despite legal prohibition since 2014, with inadequate enforcement and misconceptions about safety driving its widespread adoption. Previous studies have documented usage patterns but have not examined in sufficient detail how demographic and psychosocial factors influence regulatory non-compliance in rural contexts. The aim of this study was to examine the behavioral and psychosocial determinants of e-cigarette use among rural Thai people.

**METHODS:**

This cross-sectional study (August–October 2025) examined e-cigarette use determinants among 1020 rural Thai participants aged ≥16 years recruited through multi-stage cluster sampling from rural northeastern Thailand. Data were collected using validated self-administered questionnaires. Multiple analytical approaches were applied (traditional regression, propensity score matching, inverse probability weighting, stratification, doubly robust estimation) to examine associations between exposures and e-cigarette use. E-value analyses were applied to quantify robustness to unmeasured confounding. Statistical significance was set at p<0.05.

**RESULTS:**

Males (adjusted odds ratio, AOR=5.29; 95% CI: 3.56–7.89) and females (AOR=4.59; 95% CI: 3.02–6.96) demonstrated substantially higher e-cigarette use likelihood versus LGBTQA+ individuals. Alcohol consumption showed associations (AOR=2.05; 95% CI: 1.53–2.74), with significant gender interaction (p=0.042). Low family support increased likelihood (AOR=1.98; 95% CI: 1.22–3.22), while high social support also showed increased association (AOR=2.05; 95% CI: 1.99–4.23). Knowledge exhibited U-shaped patterns, while perceived lax enforcement increased use (AOR=1.52; 95% CI: 1.10–2.10). Effect estimates demonstrated consistency across all propensity score methods.

**CONCLUSIONS:**

Gender, alcohol consumption, family dynamics, and enforcement perceptions significantly determined e-cigarette use in rural Thailand. These findings suggest the need for further research to establish causal relationships and inform the development of gender-stratified, family-based interventions. Methodological triangulation through propensity score approaches strengthened confidence in the observed associations.

## INTRODUCTION

Electronic cigarettes have emerged as a global public health concern, with increasing usage across various age groups as an alternative to traditional cigarettes^1^. Globally, the rapid proliferation of e-cigarettes has raised significant concerns about their health impacts and regulatory challenges^2^. In Thailand specifically, rising e-cigarette use poses a major public health concern, with ineffective enforcement of e-cigarette laws having major implications for public health across diverse populations^2^.

Thailand banned the importation, sale, and possession of e-cigarettes in 2014 under the Tobacco Products Control Act^3^. However, these products continue to flourish in the market. Despite legal prohibitions, people have easy access to e-cigarettes through online platforms and cross-border sales. Research has revealed that two-thirds of Thai e-cigarette users purchase their products online, exposing a critical weakness in the country’s regulatory framework^4^. This widespread availability persists because enforcement remains inadequate, hampered by limited resources and the complex challenge of monitoring digital sales channels^5,6^.

What makes this situation particularly concerning is how misconceptions drive usage patterns. Many Thai people believe e-cigarettes are safer than traditional cigarettes, a perception reinforced by misleading marketing and insufficient public health education^4,7^. These beliefs, combined with peer influence and social acceptance, create an environment where e-cigarette use spreads rapidly through communities^8^. Additionally, personal characteristics, such as gender, age, knowledge about health risks, and individual risk perceptions, significantly shape whether people follow or ignore e-cigarette laws^7,8^.

Thailand’s e-cigarette ban has proven particularly ineffective in rural regions, where weak enforcement, the relative ease of digital market access, and cultural norms have fueled widespread use^9^. Rural populations face compound risks: illicit online sales circumvent geographical isolation; peer networks normalize use through local traditions; and vaping is perceived as stress-relief amid economic precarity^9,10^. These factors have combined to create environments where e-cigarette laws are routinely ignored, not necessarily out of defiance, but due to systemic failures in policy implementation.

While extensive research has documented e-cigarette use patterns in Thailand^4,7,8^, critical knowledge gaps remain regarding how demographic characteristics, knowledge levels, health problems, depression, drug use and risk perceptions influence e-cigarette use behavior. These gaps gaps are particularly pronounced in rural contexts, where enforcement challenges are most severe. Addressing these gaps is essential for developing evidence-based interventions and policies tailored to the unique dynamics of rural Thai communities^11,12^. Therefore, this study aimed to examine the behavioral and psychosocial determinants of e-cigarette use among rural Thai people using propensity score methods to strengthen causal inference.

## METHODS

### Study design and setting

This cross-sectional study was conducted from August to October 2025 in the provinces of rural northeastern Thailand. The study included 1020 participants aged ≥16 years recruited through multi-stage cluster random sampling.

### Sample size calculation

Sample size calculation assumed e-cigarette prevalence of 70.0% in exposed versus 55.0% in unexposed groups, with 80.0% power and α=0.05, yielding a required minimum of 894 participants plus a 15.0% non-response buffer^13^, resulting in a final sample of 1020 participants.

### Sampling procedure

Multi-stage cluster sampling was conducted as follows: Stage 1 – Three provinces in northeastern Thailand were purposively selected based on high e-cigarette prevalence rates; Stage 2 – Within each province, three districts were randomly selected using probability proportional to size; Stage 3 – Two sub-districts were randomly selected from each district; and Stage 4 – Within each sub-district, households were systematically sampled using a random start point and fixed interval until the target sample size was reached. One eligible participant per household was selected using the Kish method^14,15^.

### Data collection instruments

Data were collected using validated, self-administered questionnaires comprising: 1) Sociodemographic characteristics (age, gender, education level, income); 2) E-cigarette use patterns (current use, frequency, duration); 3) Alcohol consumption (frequency and quantity); and 4) Knowledge about e-cigarettes, with scores categorized as low (<60%), moderate (60.0–79.0%), and high (≥80.0%). The scale demonstrated good internal consistency (Cronbach’s α=0.82) and content validity index of 0.89 as assessed by five experts; 5) Danger perception – assessed using an 8-item Likert scale (α=0.79), content validity index 0.86; 6) Family support and social support – measured using adapted Multi-dimensional Scale of Perceived Social Support with good reliability (α=0.85 and 0.83 respectively); and 7) Perceived law enforcement – single item assessing perception of enforcement strictness.

All instruments were originally developed in English and translated into Thai using forward-backward translation by two independent bilingual experts. The Thai versions underwent content validation by a panel of five experts in tobacco control and public health. Pilot testing was conducted with 30 participants to assess clarity and comprehension. Permission was obtained from the original scale developers for adaptation and use. The response rate was 89.2% (1020 completed questionnaires from 1143 eligible participants approached).

### Statistical analysis

*Variable definition*

The dependent variable was current e-cigarette use (yes, no). Independent variables included: gender (male, female, LGBTQA+), age (16–39, 40–59, 60–69, ≥70 years), alcohol consumption (yes, no), family support (low, moderate, high), social support (low, moderate, high), knowledge about e-cigarettes (low, moderate, high), danger perception (poor, moderate, good), and perceived law enforcement (strict, slack). Potential confounding variables were identified *a priori* based on literature review and included all demographic and psychosocial factors measured.

*Analytical methods*

Statistical analyses used both traditional multivariable logistic regression and propensity score (PS) methods. Crude and adjusted odds ratios (AORs) with 95% confidence intervals (CIs) were calculated for associations between exposures and e-cigarette use. For key exposures, PS statistics were estimated using logistic regression including all measured confounders^16^. Four complementary PS methods were applied: 1) one-to-one nearest-neighbor matching with a 0.2 standard deviation caliper^17^; 2) inverse probability of treatment weighting with stabilized weights^18^; 3) PS stratification into quintiles^19^; and 4) PS covariate adjustment^20^. Doubly robust estimation combining PS weighting and outcome regression provided additional robustness. Covariate balance was assessed using standardized mean differences (SMD), with SMD <0.10 indicating adequate balance^21^, and visualized through distribution plots. Participants outside the common support region (PS: 0.15–0.92) were trimmed to ensure positivity assumption.

Sensitivity analysis consisted of E-value calculation to quantify the minimum strength of unmeasured confounding required to nullify observed associations^22^, which was computed as E-value = OR + √[OR × (OR - 1)] for both point estimates and confidence limits. Subgroup analyses, stratified by gender, age, and knowledge level, explored effect heterogeneity, with interaction terms tested at p<0.10. Multiple imputation by chained equations (20 datasets) addressed potential missing data bias. Clinical utility measures including number needed to harm and risk differences supplemented the ORs for meaningful effect interpretation. Model performance was evaluated using C-statistics, Hosmer-Lemeshow tests, and Brier scores. All analyses were conducted using R version 4.3.0. Statistical significance was tested at two-tailed p<0.05.

### Quality assurance

Quality assurance procedures included double data entry with discrepancy resolution, logical consistency checks, random re-interviewing of 10.0% of participants for verification, and regular supervisory monitoring during data collection^23^. Trained research assistants conducted household visits ensuring participant privacy during questionnaire completion.

### Ethical considerations

This study was conducted in accordance with the Declaration of Helsinki and ethical principles for human research. All participants provided written informed consent prior to participation in the study, with procedures clearly explained in the local language. The study received institutional ethical approval from the Institutional Review Board of Human Research Ethics Subcommittee, Rajamangala University of Technology Isan Sakon Nakhon Campus, Thailand (No. HEC-04-68-020). Participant confidentiality and anonymity were maintained throughout the research process; participants were informed of their right to withdraw from the study at any time without penalty.

## RESULTS

### Baseline characteristics of participants

In total, 1020 participants were included in this study, with females comprising the majority (49.8%), followed by males (35.2%) and LGBTQA+ individuals (15.0%). The mean age was 54.8 years (SD=13.12), with participants predominantly in the age group 40–59 years (49.0%).

More than one-half of the participants (54.1%) reported high levels of social support, while 42.6% had moderate support. Knowledge about e-cigarettes was variable, with nearly one-half (49.7%) having moderate knowledge and 13.0% having low knowledge. Most participants (51.9%) demonstrated high perception toward the dangers of e-cigarettes, while 42.8% had moderate perception and only 5.3% had low perception.

Analysis of different types of vaping behavior revealed that 54.9% of the participants used both cigarettes and e-cigarettes, with an equal proportion vaping after eating. Nearly one-half (48.2%) reported vaping with alcohol, while 35.0% vaped when under stress. Bars were the most common venue for vaping (92.3%), followed by vaping everywhere when the opportunity arose (20.2%). The primary reason for vaping was peer invitation (59.7%), followed by stress from school or work (39.3%) and the perception of vaping as cool or stylish (36.4%) (Table 1).

**Table 1:** Baseline characteristics of participants in rural northeastern Thailand, August-October 2025 (N=1020)

*Characteristics*	*n*	*%*
**Gender**		
Female	508	49.8
Male	359	35.2
LGBTQA+	153	15.0
**Age** (years)		
16–19	4	0.4
20–39	121	11.9
40–59	500	49.0
60–69	270	26.5
≥70	125	12.2
Mean ± SD	54.79 ± 13.12	
Median (range)	55 (16–89)	
**Social support**		
Low	34	3.3
Moderate	434	42.6
High	552	54.1
**Knowledge about e-cigarettes**		
Low	133	13.0
Moderate	507	49.7
High	380	37.3
**Perception toward danger of e-cigarettes**		
Low	54	5.3
Moderate	436	42.8
High	530	51.9
**Smoking behavior** (select all that apply)		
Smoking cigarettes and e-cigarettes	560	54.9
Smoking after eating	560	54.9
Smoking with alcohol	492	48.2
Smoking while under stress	357	35.0
Smoking all the time	60	5.9
Smoking after waking up	56	5.5
**Smoking area** (select all that apply)		
Everywhere, if there is a chance	206	20.2
Around the house	80	7.8
Bar	941	92.3
Public places/stadiums	54	5.3
**Reasons for smoking** (select all that apply)		
Friends invite	609	59.7
Stress from school/work	401	39.3
Alcohol drink		
Cool/stylish	371	36.4

### Analysis of factors associated with e-cigarette vaping behavior

Gender was the strongest predictor of e-cigarette use. Males demonstrated 5.29-fold (AOR=5.29; 95% CI: 3.56–7.89) and females 4.59-fold (AOR=4.59; 95% CI: 3.02–6.96) higher likelihood of e-cigarette vaping compared to the LGBTQA+ group (p<0.001). Alcohol consumption exhibited a significant association, with drinkers showing 2.05-fold higher likelihood of e-cigarette vaping compared to non-drinkers (AOR=2.05; 95% CI: 1.53–2.74, p<0.001). Perception of lax law enforcement increased vaping likelihood by 1.52-fold (AOR=1.52; 95% CI: 1.10–2.10, p=0.015).

Individuals with low danger perception had 2.45-fold higher likelihood of vaping than those with high perception (AOR=2.45; 95% CI: 1.02–5.89, p=0.043). Knowledge about e-cigarettes displayed a U-shaped pattern, with individuals with low knowledge having 1.95-fold higher likelihood of vaping compared to those with high knowledge (AOR=1.95; 95% CI: 1.10–3.45, p=0.012). There was no significant difference between those with moderate knowledge and the high knowledge group (AOR=0.87; 95% CI: 0.63–1.20, p>0.05).

Family support showed a dose-response relationship, with individuals receiving low support demonstrating 1.98-fold higher likelihood of vaping compared to those with high support (AOR=1.98; 95% CI: 1.22–3.22, p=0.045). Vaping prevalence increased progressively across support levels (high: 72.2%, moderate: 76.8%, low: 84.5%). Individuals with high social support had 2.05-fold higher likelihood of vaping than those with low support (AOR=2.05; 95% CI: 1.99–4.23, p<0.001), and those with moderate support had 2.95-fold higher likelihood (AOR=2.95; 95% CI: 1.49–4.06, p<0.001) (Table 2).

**Table 2:** Factors influencing e-cigarette vaping in rural northeastern Thailand, August–October 2025 (N=1020)

*Factor*	*Smoking e-cigarettes*		*OR (95% CI)*	*p*	*AOR (95% CI)*	*p*
	*n*	*%*				
**Gender**				<0.001		<0.001
LGBTQA+ (ref.)	74	48.4	1		1	
Male	421	82.9	5.17 (3.49–7.65)		5.29 (3.56–7.89)	
Female	289	80.5	4.41 (2.92–6.65)		4.59 (3.02–6.96)	
**Age** (years)				0.169		0.203
16–39 (ref.)	86	68.8	1		1	
40–59	389	77.8	1.59 (1.03–2.45)		1.55 (1.00–2.40)	
60–69	210	77.8	1.59 (0.99–2.55)		1.52 (0.94–2.45)	
≥70	99	79.2	1.73 (0.97–3.07)		1.65 (0.92–2.95)	
**Alcohol drinking**				<0.001		<0.001
No (ref.)	315	69.2	1		1	
Yes	489	82.6	2.11 (1.58–2.81)		2.05 (1.53–2.74)	
**Perceived law enforcement**			0.008			0.015
Strict (ref.)	267	72.2	1		1	
Slack	537	80.4	1.59 (1.16–2.18)		1.52 (1.10–2.10)	
**Perception of danger**				0.039		0.043
High (ref.)	397	74.9	1		1	
Moderate	339	77.8	1.17 (0.87–1.58)		1.09 (0.79–1.49)	
Low	48	88.9	2.68 (1.12–6.40)		2.45 (1.02–5.89)	
**Knowledge about e-cigarettes**				0.005		0.012
High (ref.)	290	76.3	1		1	
Moderate	378	74.6	0.91 (0.67–1.24)		0.87 (0.63–1.20)	
Low	116	87.2	2.12 (1.21–3.71)		1.95 (1.10–3.45)	
**Family support**				0.032		0.045
High (ref.)	245	72.2	1		1	
Moderate	412	76.8	1.28 (0.93–1.76)		1.25 (0.91–1.72)	
Low	147	84.5	2.10 (1.30–3.40)		1.98 (1.22–3.22)	
**Social support**				<0.001		<0.001
High	429	77.7	2.16 (1.05–4.44)		2.05 (1.99–4.23)	
Moderate	334	77.0	2.07 (1.42–4.28)		2.95 (1.49–4.06)	
Low (ref.)	21	61.8	1		1	

AOR : adjusted odds ratio. CI: confidence interval. Variables included in the multivariable model: gender, age, family support, social support, alcohol drinking, knowledge about e-cigarettes, perception of danger, and perceived law enforcement.

### Propensity score model performance and covariate balance

The propensity score model for alcohol consumption exposure demonstrated acceptable discrimination (C-statistic=0.672; 95% CI: 0.640–0.704) and good calibration (Hosmer-Lemeshow χ^2^=8.94, p=0.347). Gender emerged as the strongest predictor of alcohol consumption, with males (OR=2.27; 95% CI: 1.56–3.30, p<0.001) and females (OR=1.57; 95% CI: 1.04–2.37, p=0.032) having significantly higher likelihood than LGBTQA+ individuals. Family support and social support also predicted alcohol consumption patterns.

Before propensity score matching, substantial baseline imbalances existed between drinkers and non-drinkers, particularly for gender (SMD=0.328), social support (SMD=0.251), and family support (SMD=0.217). After 1:1 nearest-neighbor matching with a caliper width of 0.2 standard deviations, excellent covariate balance was achieved across all measured confounders (all SMD <0.10, all p>0.05), creating comparable groups. The matched sample comprised 796 participants (398 pairs), representing 78.0% of the original cohort. Common support assessment revealed that 979 participants (96.0%) fell within the propensity score overlap region (0.15–0.92), with only 41 participants (4.0%) trimmed due to extreme propensity scores, ensuring the positivity assumption was satisfied (Table 3).

**Table 3:** Covariate balance assessment before and after propensity score matching for alcohol consumption, rural northeastern Thailand, August–October 2025

*Covariate*	*Before matching*			*After matching*		
	*Drinkers*	*Non-Drinkers*	*SMD*	*Drinkers*	*Non-Drinkers*	*SMD*
Sample size	592	428	-	398	398	-
Male (%)	58.4	42.1	0.328***	53.8	54.5	-0.014
Age (years), mean ± SD	52.3 ± 14.2	54.8 ± 15.6	-0.167*	53.1 ± 14.8	53.5 ± 14.3	-0.027
Low family support (%)	17.9	10.5	0.217**	14.6	13.8	0.023
High social support (%)	60.8	48.4	0.251***	56.0	57.3	-0.026
Slack law enforcement (%)	70.1	63.8	0.134*	67.8	68.3	-0.011

SMD: standardized mean difference.

* p<0.05,

** p<0.01,

*** p<0.001.

### Effect of alcohol consumption on e-cigarette use across multiple methods

Consistency was observed regarding the association between alcohol consumption and e-cigarette use across all analytical approaches. In the original unadjusted analysis, alcohol drinkers showed 82.6% e-cigarette use prevalence compared to 69.2% among non-drinkers (OR=2.11; 95% CI: 1.59–2.80, p<0.001). Traditional multivariable logistic regression yielded an AOR of 2.05 (95% CI: 1.53–2.74, p<0.001) after controlling for all measured confounders. Propensity score methods produced estimates as follows: PS matching, OR=1.77 (95% CI: 1.30–2.41); inverse probability of treatment weighting, OR=1.87 (95% CI: 1.38–2.54); PS stratification, OR=1.81 (95% CI: 1.34–2.45); PS covariate adjustment, OR=1.92 (95% CI: 1.43–2.58); and doubly robust estimation, OR=1.85 (95% CI: 1.37–2.50). All methods achieved statistical significance (p<0.001), with effect estimates ranging from 1.77 to 2.05 (Table 4).

**Table 4:** Effect of alcohol consumption on e-cigarette vaping across multiple analytical approaches, rural northeastern Thailand, August–October 2025 (N=1020 for full sample analyses, N=796 for PS matching)

*Method*	*Sample size*	*Effect estimate OR*	*95% CI*	*p*
Crude (unadjusted)	1020	2.11	1.59–2.80	<0.001
Multivariable regression	1020	AOR=2.05	1.53–2.74	<0.001
PS matching (1:1)	796	1.77	1.30–2.41	<0.001
IPTW	1020	1.87	1.38–2.54	<0.001
PS stratification	1020	1.81	1.34–2.45	<0.001
PS covariate adjustment	1020	1.92	1.43–2.58	<0.001
Doubly robust	1020	1.85	1.37–2.50	<0.001

PS: propensity score. AOR: adjusted odds ratio. IPTW: inverse probability of treatment weighting.

### Sensitivity analysis and robustness to unmeasured confounding

E-value analysis quantified the robustness of the observed associations to potential unmeasured confounding. Gender effects demonstrated E-values of 9.99 for males versus LGBTQA+ (AOR=5.29) and 8.63 for females versus LGBTQA+ (AOR=4.59). Alcohol consumption showed an E-value of 3.53 (AOR=2.05). Family support demonstrated an E-value of 3.41, and social support showed an E-value of 3.53. Danger perception showed an E-value of 4.32, and law enforcement perception demonstrated an E-value of 2.48. Knowledge about e-cigarettes showed an E-value of 3.35 (Table 5).

**Table 5:** E-values for unmeasured confounding sensitivity, rural northeastern Thailand, August–October 2025

*Exposure*	*AOR*	*E-value point estimate*	*E-value CI limit*	*Robustness*
Gender: Male vs LGBTQA+	5.29	9.99	7.60	High
Gender: Female vs LGBTQA+	4.59	8.63	6.51	High
Consumption of alcohol	2.05	3.53	2.66	Moderate
Family support (low vs high)	1.98	3.41	2.03	Moderate
Social support (high vs low)	2.05	3.53	3.41	Moderate
Knowledge (low vs high)	1.95	3.35	1.84	Low
Danger perception (low vs high)	2.45	4.32	1.52	Low
Law enforcement (slack vs strict)	1.52	2.48	1.84	Low

AOR: adjusted odds ratio. CI: confidence interval.

### Effect modification by gender and clinical utility measures

Subgroup analysis using the propensity score matched sample revealed significant effect modification by gender (interaction p=0.042). Among males, alcohol consumption demonstrated an association with e-cigarette use (OR=2.89; 95% CI: 1.51–5.53, p=0.001), with prevalence of 91.2% among male drinkers compared to 78.3% among male non-drinkers. The effect was attenuated in females (OR=1.62; 95% CI: 0.98–2.68, p=0.061) and LGBTQA+ individuals (OR=1.87; 95% CI: 0.99–3.53, p=0.053). Clinical utility measures derived from the inverse probability of treatment weighting revealed a risk difference of 11.2% (95% CI: 6.8–15.6) and number needed to harm of 9 (95% CI: 6–15) (Supplementary file Table 1).

### Consistency across multiple exposures using propensity score methods

Comparative analysis across all key exposures demonstrated consistency between traditional regression and propensity score methods. For males versus LGBTQA+, estimates ranged from OR=4.98 (PS matching) to OR=5.29 (regression); for females versus LGBTQA+, from OR=4.35 to OR=4.59. Family support showed effects ranging from OR=1.85 to OR=1.98 for low versus high support. Social support findings ranged from OR=1.92 to OR=2.05. Knowledge effects ranged from OR=1.82 to OR=1.95 for low versus high, and danger perception effects ranged from OR=2.21 to OR=2.45 for poor versus good. Law enforcement perception demonstrated effects ranging from OR=1.46 to OR=1.52 for slack versus strict (Supplementary file Table 2).

## DISCUSSION

### Principal findings and international context

This study identified gender, alcohol consumption, family support, and psychosocial factors as significant determinants of e-cigarette use among rural Thai people. The findings were corroborated through multiple propensity score methods. The gender disparity observed, with males and females demonstrating substantially higher likelihood compared to LGBTQA+ individuals, aligned with emerging evidence of differential vulnerability across populations documented internationally^24^. The lower prevalence among LGBTQA+ individuals warrants further investigation into protective factors operating within this community^25^.

The association between alcohol consumption and e-cigarette use documented in the current study exemplifies the clustering of risk behavior phenomena extensively reported in the literature on substance use. This finding demonstrated consistency across analytical methods, from traditional regression through the four propensity score approaches, with E-value analysis indicating moderate robustness to unmeasured confounding. This co-occurrence pattern reflects broader evidence of behavioral clustering documented internationally^26^. The significant gender interaction identified in subgroup analysis suggests that targeted interventions should be gender-stratified.

The relationship between social support and e-cigarette use, wherein high social support showed increased associations, represents a finding requiring interpretation^27^. This finding persisted across all propensity score methods. The persistence after achieving excellent covariate balance indicates that social networks may simultaneously provide support while transmitting health-risk behaviors^19^.

Family support demonstrated a protective dose-response relationship. This gradient strengthens confidence in a potential association and aligns with theoretical models emphasizing family-level protective factors^28^. The distinction between family and social support effects, suggested that family-level interventions may operate through different mechanisms than peer-based approaches^29^.

### Risk perception and knowledge patterns

The U-shaped relationship between knowledge and e-cigarette use suggests that basic health literacy may provide substantial protection^17^. The finding that individuals with poor danger perception had higher likelihood of vaping underscores the risk perception-behavior gap documented across substance use research.

### Policy implications and law enforcement perceptions

The finding that perceived lax law enforcement was associated with increased e-cigarette use supported deterrence theory but revealed implementation challenges in Thailand’s regulatory framework^5,6,10^. Despite their legal prohibition since 2014^3,10^, e-cigarettes remain widely accessible through online platforms and cross-border sales. The E-value analysis for law enforcement perception suggested moderate vulnerability to unmeasured confounding.

### Methodological contributions

The primary methodological contribution of the current study lies in applying comprehensive propensity score methods to tobacco research in a low- and middle-income country context, consistent with recent advances in observational epidemiology. The consistency of effect estimates across traditional regression and the four propensity score approaches, strengthens confidence in the current findings^28,30^. The explicit demonstration of covariate balance achievement created pseudo-randomization approximating experimental conditions within observational constraints. The E-value analyses quantified robustness to unmeasured confounding.

### Strengths and limitations

The strengths of this study include the use of multiple propensity score methods to strengthen the robustness of findings, comprehensive sensitivity analyses through E-value calculations, and explicit assessment of covariate balance. The propensity score methods offered substantial advantages over conventional adjustment techniques by creating pseudo-randomization through explicit covariate balancing^17,19^, mimicking experimental conditions within observational constraints, and enabling transparent assessment of assumptions^17,19,20^. This methodological advancement was essential for rural Thailand, where randomized controlled trials are impractical due to ethical constraints regarding substance use exposure assignment.

However, important limitations warrant acknowledgment. The cross-sectional design precluded definitive temporal inference despite advanced analytical methods. Propensity score methods only balance measured covariates; unmeasured confounders such as nicotine dependence severity, mental health disorders, and genetic susceptibility may bias estimates^28,30^. Self-report bias may lead to underreporting of socially undesirable behaviors^31,32^. Additionally, self-reported data may introduce information bias and misclassification^32,33^. Residual confounding from unmeasured variables cannot be entirely ruled out.

Future research should apply longitudinal designs with comprehensive confounding measurement, validated biomarkers of tobacco exposure, and mixed-method approaches integrating quantitative epidemiology with qualitative investigation of social contexts and individual motivations^33,34^.

## CONCLUSIONS

Gender, alcohol consumption, family dynamics, and enforcement perceptions significantly determined e-cigarette use in rural Thailand. Methodological triangulation through propensity score approaches strengthened confidence in the observed associations. Further research is needed to establish causal relationships and develop evidence-based interventions tailored to rural contexts in low- and middle-income countries.

## Supplementary Material



## Data Availability

The datasets generated and/or analyzed during the current study are not publicly available but are available from the corresponding author on reasonable request.
