# Supplementary material for: Behavioral and psychosocial predictors of e-cigarette use in rural Thailand: A cross-sectional propensity score-matched study
Source: Tob Induc Dis. 2026 May 8;24:10.18332/tid/218287. doi: 10.18332/tid/218287 (PMC13154162; doi:10.18332/tid/218287)
Supplement: Supplementary file 1 [file TID-24-56-s1.pdf]

Supplementary file

Table 1. *Gender-stratified effects of alcohol consumption on e-cigarette vaping and clinical utility measures, rural northeastern Thailand, August–October 2025*

Analysis	Estimate	95% CI	P-value
Subgroup Analysis (PS Matched Sample)			
Male	OR: 2.89	1.51-5.53	0.001
Female	OR: 1.62	0.98-2.68	0.061
LGBTQA+	OR: 1.87	0.99-3.53	0.053
Interaction Test			0.042
Clinical Utility Measures (IPTW)			
Risk Difference	11.2%	6.8%-15.6%	<0.001
Number Needed to Harm	9	6-15	-

OR = Odds Ratio; CI = Confidence Interval; PS = Propensity Score; IPTW = Inverse Probability of Treatment Weighting.

Table 2. *Comparative effect estimates across key exposures for e-cigarette vaping, rural northeastern Thailand, August–October 2025*

Exposure	PS Matching OR (95% CI)	IPTW OR (95% CI)	Regression AOR (95% CI)
Alcohol: Yes vs No	1.77 (1.30-2.41)	1.87 (1.38-2.54)	2.05 (1.53-2.74)
Gender: Male vs LGBTQA+	4.98 (3.21-7.72)	5.12 (3.41-7.69)	5.29 (3.56-7.89)
Gender: Female vs LGBTQA+	4.35 (2.76-6.86)	4.51 (2.96-6.87)	4.59 (3.02-6.96)
Family Support: Low vs High	1.85 (1.09-3.14)	1.91 (1.16-3.15)	1.98 (1.22-3.22)
Social Support: High vs Low	1.92 (0.87-4.24)	1.98 (0.93-4.21)	2.05 (1.99-4.23)
Knowledge: Low vs High	1.82 (0.97-3.41)	1.88 (1.02-3.47)	1.95 (1.10-3.45)
Danger Perception: Poor vs Good	2.21 (0.87-5.62)	2.32 (0.94-5.73)	2.45 (1.02-5.89)
Law Enforcement: Slack vs Strict	1.46 (1.02-2.09)	1.49 (1.06-2.10)	1.52 (1.10-2.10)

PS = Propensity Score; OR = Odds Ratio; AOR = Adjusted Odds Ratio; CI = Confidence Interval; IPTW = Inverse Probability of Treatment Weighting.

© 2026 Sorasit J. et al.
